# Bacterial Communities of Three Saline Meromictic Lakes in Central Asia

**DOI:** 10.1371/journal.pone.0150847

**Published:** 2016-03-02

**Authors:** Bayanmunkh Baatar, Pei-Wen Chiang, Denis Yu Rogozin, Yu-Ting Wu, Ching-Hung Tseng, Cheng-Yu Yang, Hsiu-Hui Chiu, Bolormaa Oyuntsetseg, Andrey G. Degermendzhy, Sen-Lin Tang

**Affiliations:** 1 Molecular and Biological Agricultural Sciences Program, Taiwan International Graduate Program, Academia Sinica, Taipei, Taiwan; 2 Biodiversity Research Center, Academia Sinica, Taipei, Taiwan; 3 Graduate Institute of Biotechnology, National Chung-Hsing University, Taichung, Taiwan; 4 Institute of Biophysics SB RAS, 660036 Krasnoyarsk, Russia; 5 National Pingtung University of Science and Technology, Pingtung, Taiwan; 6 School of Art and Sciences, National University of Mongolia, Ulaanbaatar 14201, Mongolia; 7 Biotechnology Center, National Chung-Hsing University, Taichung, Taiwan; National Cheng-Kung University, TAIWAN

## Abstract

Meromictic lakes located in landlocked steppes of central Asia (~2500 km inland) have unique geophysiochemical characteristics compared to other meromictic lakes. To characterize their bacteria and elucidate relationships between those bacteria and surrounding environments, water samples were collected from three saline meromictic lakes (Lakes Shira, Shunet and Oigon) in the border between Siberia and the West Mongolia, near the center of Asia. Based on in-depth tag pyrosequencing, bacterial communities were highly variable and dissimilar among lakes and between oxic and anoxic layers within individual lakes. *Proteobacteria*, *Bacteroidetes*, *Cyanobacteria*, *Actinobacteria* and *Firmicutes* were the most abundant phyla, whereas three genera of purple sulfur bacteria (a novel genus, *Thiocapsa* and *Halochromatium)* were predominant bacterial components in the anoxic layer of Lake Shira (~20.6% of relative abundance), Lake Shunet (~27.1%) and Lake Oigon (~9.25%), respectively. However, few known green sulfur bacteria were detected. Notably, 3.94% of all sequencing reads were classified into 19 candidate divisions, which was especially high (23.12%) in the anoxic layer of Lake Shunet. Furthermore, several hydro-parameters (temperature, pH, dissolved oxygen, H_2_S and salinity) were associated (P< 0.05) with variations in dominant bacterial groups. In conclusion, based on highly variable bacterial composition in water layers or lakes, we inferred that the meromictic ecosystem was characterized by high diversity and heterogenous niches.

## Introduction

Meromictic lakes are unique ecosystems with water profiles strongly stratified chemically and incompletely mixed over multi-year intervals [1–4]. Approximately 200 saline meromictic lakes have been discovered, corresponding to <1% of all known lakes [2]. The water columns of these lakes are commonly stratified into two major zones, the mixolimnion (*i*.*e*., upper, oxygenic water) and monimolimnion (*i*.*e*., bottom, anoxic water), which are frequently separated by a chemocline (transition zone) [1, 2, 5–8]. Stratification generally results from stable gradients in physiochemical factors in these lakes [9, 10].

A meromictic lake is a good model for studying microbial ecology [4, 7, 11], due to long-term vertical stratification of bacterial populations and clearly separated, physically stable water mass compartments [1, 2, 7, 9]. These lakes are reported to have unique bacterial communities at various depths [4, 10, 12, 13], with cyanobacteria often dominating the oxic surface layer [14, 15], anaerobic phototrophic bacteria occupying the chemocline, and sulfide oxidizers and reducers tightly linked to anoxic deep layers [4, 16–18].

These lakes facilitate studying ecological-function interactions between bacteria and specific biogeochemical processes [10, 19]. However, compared to freshwater and marine environments that have been more thoroughly explored, relatively little is known regarding microbial ecology of meromictic lakes [13, 20]. In the last decade, several studies have focused on isolation and characterization of phototrophic bacteria [3, 13, 21], particularly their composition in chemoclines, which frequently contain abundant purple sulfur bacteria (PSB) and green sulfur bacteria (GSB) [2, 6, 22–24]. However, there are apparently only five comprehensive surveys of bacterial communities of vertically stratified water zones of meromictic lakes [4, 8, 14, 19, 25], of which three involve lakes at the north or south poles.

Polar meromictic lakes were formed by isostatic uplift, which caused isolation from seawater followed by subsequent inflow of melt-water and the last de-glaciation [4, 14, 25]. Furthermore, year-round ice covers a very stable water column by conferring protection from wind-driven mixing and temperature differentials [14, 15]. Meromictic lakes are most common in coastal areas [8, 14, 18, 26] (especially in the Arctic and Antarctica), whereas very few have been identified in land-locked areas distant from the ocean [10, 20]. Lakes Shira, Shunet and Oigon are saline meromictic lakes located in the landlocked steppes around the geographic center of Asia (Kyzyl), ~2500 km from any coastline. Lake Shira (9.3 x 5.3 km, 35.9 km^2^ and 24 m deep) and Lake Shunet (1.2 x 0.4 km, 0.47 km^2^, and 6.2 m) are two of only three saline meromictic lakes identified in Siberian Russia [9, 27], whereas Lake Oigon (15.5 x 7 km, 61.3 km^2^, 9 m), newly discovered during this study, is the first meromictic lake known in Mongolia.

The limnological characteristics of Lakes Shira and Shunet, including ecological modeling, characterization of environmental factors, elemental cycles with temporal and spatial variations, and surveys of flora and fauna, have been reported [9, 20, 21, 28–30]. Physicochemical properties, water color, etc., were direct evidence for stratification. However, bacterial communities in these lakes have not been well characterized (coupled with a complete absence of studies for Lake Oigon in western Mongolia). In Lake Shira and Lake Shunet, bacterial surveys have only focused on a few predominant groups, such as PSB (related to *Lamprocystis purpurea*, *Thiocapsa* and *Halochromatium* species) and GSB (related to *Prosthecochloris vibrioformis*) in the chemocline [10, 29, 31, 32]. Abundance and seasonal dynamics of PSB in the two lakes have been characterized by pigment analysis, cultivation, and microscopic analysis [31, 33]. Intriguingly, Lake Shunet had an unexpectedly high density of PSB (up to 1.8±0.4 x 10^8^ cells/mL in the chemocline [10, 32]), comparable to Lake Mahoney (4 x 10^8^ cells/mL), which had the highest density of PSB reported in a meromictic lake [18, 29]. Predominant bacteria in chemoclines of these lakes were characterized using 16S rRNA genes amplified by PCR and visualized by denaturing gradient gel electrophoresis [10]. However, such fragmented information regarding bacterial communities did not adequately characterize relationships between bacteria and their surrounding environment. Therefore, a comprehensive survey is indicated.

The goals of this study were to characterize and compare bacterial communities throughout the water column and determine associations between environmental parameters and bacterial populations among individual layers of these meromictic lakes. Bacterial community composition and biodiversity of the three lakes were determined by 454-pyrosequencing the V1/V2 hyper-variable regions of the 16S rRNA gene. There were clear similarities and differences in bacterial community structure among lakes, as well as unique bacterial profiles in distinct water layers. Furthermore, associations between bacterial community and environmental parameters were explored. Finally, this was the first report to characterize the bacterial community and diversity in Lake Oigon.

## Materials and Methods

No specific permissions were required to collect water samples and hydro-parameter data in Lake Shira (54°30′N and 90°10′E), Lake Shunet (54°25′N and 90°13′E) and Lake Oigon (49°16′N and 90°58′E). The study areas were not carried out on private land, a protected area or a national park. Proposals were approved by the committee of the Taiwan-Russian Joint Project (NSC99-2923-B-001-001-MY3 and NSC 102-2923-B-001-004) and Taiwan-Mongolian Joint Project (NSC101-2923-B-001-003-MY3) from the National Sciences Council of Taiwan and the Russian Foundation for Basic Research and Ministry of Education, Culture and Sciences of Mongolia. In addition, field and lab studies did not involve any animal husbandry, nor any protected or endangered biological species.

### Description of study sites and sampling procedures

**Fig 1:**
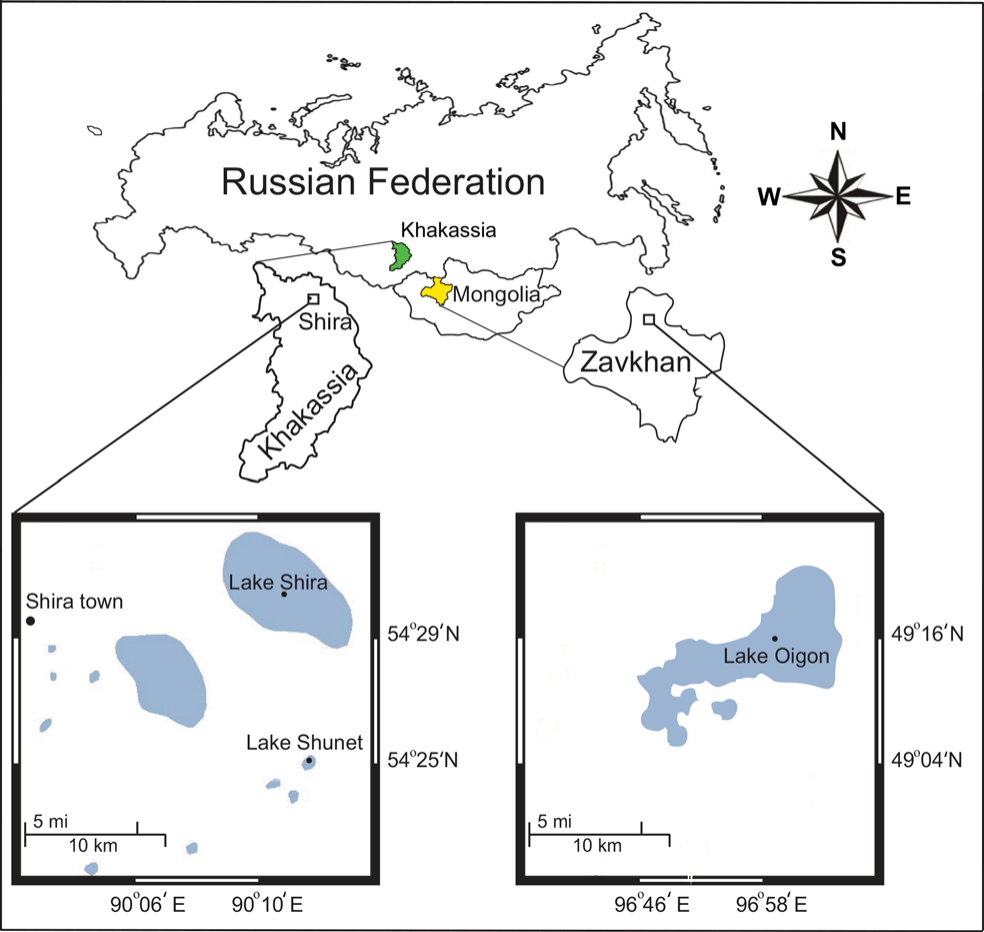
Location of sampling sites; Lake Shira and Lake Shunet in Khakassia, Russia and Lake Oigon in western Mongolia.

Lakes Shira, Shunet and Oigon are meromictic saline lakes in the steppe area of the Republic of Khakassia, Siberia (Russian Federation) and western Mongolia (Fig 1) [20, 28]. Water columns in these lakes are stratified into two main layers: oxic (mixolimnion) and anoxic (chemocline and monimolimnion) [9, 10]. Thirty-six water samples were collected from depths [34] of oxic (1–12 m) and anoxic (14–23 m) layers in Lake Shira, oxic (1–4 m) and anoxic (5–6 m) layers in Lake Shunet, and oxic (0–7 m) and anoxic (7.75–9 m) layers in Lake Oigon (S1 Table). Samples consisted of 6 L of lake water vertically collected (Years 2010–2013) from each sampling depth with a handmade vacuum deep-water sampler (S1 Fig). This device has a very similar function to the composite high-precision stratification sampler. Water samples were retained in sterile 6 L containers, directly transported to the field station (located on the shore of each lake), initially filtered (10 μm plankton net) and sequentially run through a Millipore-Pellicon TFF system (0.22 μm filter membrane) to collect retentates. Bacteria were obtained from retentates by filtering through 0.22 μm polycarbonate membrane filters using a vacuum pump in the field station. These membranes were subsequently air dried under a laminar flow at room temperature in the field station for 15 min, then sealed in separate sterile bags until arrival at the laboratory. The dried membranes were stored at 4°C for 48–72 h in the laboratory until DNA was extracted.

### Analysis of physical and chemical parameters

Vertical profiles for temperature, specific conductance, dissolved oxygen, pH, and salinity were measured with Hanna HI 9828 and YSI 6600 submersible profilers (Yellow Springs, Dayton, OH, USA). Conductivity readings of *in situ* temperatures (*C*_*t*_) were standardized to specific conductance at 25^○^C using *K*_25_ = *C*_*t*_ *× (1+0*.*0204× (T-25))*^*-1*^, where *T* is the *in-situ* temperature in degrees Celsius (YSI). The relationship between salinity and conductivity for a lake is: *S (g L*^*-1*^*) = 1*.*117 K*_*25*_*− 7*.*9716*. Conductivity sensors were calibrated against 3 M KCl (Hydrolab, YSI) and conducted before each survey. For sulphide determination, subsamples were fixed with zinc acetate and determined by a colorimetric method [35]. The following analyses were all conducted as previously described [36]. Phosphorus (P) and ammonia (NH_4_^+^) were determined using routine ammonium molibdate and the Nesserilization method, respectively. Regarding the latter, NO_2^-^_ was determined through formation of purple dye produced by the reaction of NO_2^-^_ with Gries reagent, as NO_3^-^_ was converted into NO_2^-^_ by reduction with the Cd redactor. Furthermore, SO_4_^2-^ was measured with a standard barium chloride method.

### DNA extraction, amplicon library generation, and pyrosequencing

Total DNA was extracted using the cetyltrimethylammonium bromide (CTAB) method [37]. Bacterial amplicon libraries of the V1/V2 hypervariable region of the 16S rRNA gene were produced with universal primers: 27F (5’-AGAGTTTGATCMTGGCTCAG-3’) and 341R (5’-CTGCTGCCTCCCGTAGG-3’). The PCR reactions occurred in a total volume of 50 μL per sample containing 2.5 U *TaKaRa EX taq*^TM^ HS, 5 μL of 10X *EX taq* Buffer, 200 μM of dNTPs, 0.2 μM of each primer, and 2~5 μg diluted template DNA (final concentration 100 ng/μL). The PCR program was initiated by denaturation at 94°C for 3 min, followed by 30 cycles of 94°C for 20 s, 52°C for 20 s, and 72°C for 20 s, with a final step at 72°C for 2 min, and then cooling (4°C). The PCR products were verified using 1% agarose gel electrophoresis with 1X TE buffer and SYBR® Green I. Expected-sized products (~300 bp) were cut from the gel and purified using a QIAEX II Gel Extraction Kit (QIAGEN, Valencia, CA, USA). Purified DNAs were quantified using a NanoDrop spectrophotometer (Thermo Scientific, Vantaa, Finland). In a second round of PCR amplification, unique four nucleotide sample-specific 36 barcodes were added to 5´ends of 27F and 341R primers for each sample (S2 Table). DNA tagging PCR (DT-PCR) was used to fuse unique tags to each of the PCR products, which was conducted as described [38].

The PCR mixture contained 2.5 U *TaKaRa EX taq*^TM^ HS (TaKaRa Bio, Otsu, Japan), 5 μL of 10X *EX taq* Buffer, 200 μM of dNTPs, 0.4 μM of each barcoded primer, and 100 ng V1/V2 amplicon in a final volume of 50 μL. The PCR program was initiated by denaturation at 94°C for 3 min, followed by five cycles of 94°C for 20 s, 52°C for 20 s, and 72°C for 20 s, with a final step at 72°C for 2 min and then cooling at 4°C. The PCR products were verified using 1% agarose gel electrophoresis with 1X TE buffer and SYBR® Green I. Expected-sized products (~300 bp) were cut from the gel and purified using a QIAEX II Gel Extraction Kit (QIAGEN, Valencia, CA, USA). Purified DNAs were quantified using a NanoDrop spectrophotometer (Thermo Scientific, Vantaa, Finland). Finally, PCR products were pooled together and a 200 ng mixture of tagged V1-V2 regions was subjected to pyrosequencing (Roche GS454 FLX Titanium, Mission Biotech, Taipei, Taiwan).

### Sequence analysis

Analysis of raw 454 pyrosequencing data were processed using MOTHUR v.1.33.3 [39]. Raw sequence data were quality-trimmed with the flowing pipeline: sequence length outside the 250–350 bp range; sequence read having ambiguous nucleotides (N); average quality score <27; homopolymer length >6; and mismatched primers and incomplete barcodes removed prior to further analysis (S2 Fig). A total of 139,788 qualified sequences were retained and grouped into various samples according to barcode using an in-house sorting script (http://tanglab.csie.org/scripts).

After quality trimming and sorting, chimeric sequences were detected using UCHIME [40] and removed to retain high-quality reads.

Qualified and non-chimeric sequences were analyzed with the UPARSE (http://drive5.com/uparse/) to generate operational taxonomic units (OTUs) at 97% similarity level and classified with taxonomic labels from SILVA-ngs pipeline [41]. All singleton OTUs, chloroplasts and unclassified sequences ("No Relative" by SILVA-ngs) were excluded from further analyses as potential noise.

On a per-sample basis, multiple sequence alignment was generated with MUSCLE (http://drive5.com/muscle/) [42] and the corresponding distance matrix was calculated with the dnadist function in PHYLIP package v3.69 (http://evolution.genetics.washington.edu/phylip.html). Based on the distance matrix, MOTHUR v.1.33.3 [39] was used to generate OTUs at 97% similarity level and estimate Shannon-Weaver diversity [43], Simpson’s similarity index [44], Chao1 (bias-corrected species richness estimator) [45], and ACE (non-parametric Abundance-based Coverage Estimator) [46].

### Data analyses

The OTUs from each sample were used for further analyses. The relative abundance of each OTU was log-transformed before being subjected to non-Metric Multidimensional Scaling (nMDS), based on the Bray-Curtis distance matrix using Primer 6 software (PRIMER-E package, Version 6; Plymouth Marine Laboratory, Plymouth, UK).

Hydro-parameters were checked for collinearity using Spearman’s Rank correlation, and a set of parameters with low correlations (R<0.5) were selected for Canonical Correspondence Analysis (CCA) to explore contributions of environmental parameters to community structure. Furthermore, a heat map was constructed based on relative abundance of each OTU. The CCA and heat map were analyzed in R (http://www.R-project.org/; R Development Core Team). Correlations between individual hydro-parameters and community structure indices were examined using Pearson’s correlation in JMP software (http://www.jmp.com/). A candidate division OD1 profile was constructed using OD related sequences from the Lake Shunet, pooled for two layers. The relative abundance of OD1 reads in each layer was normalized by sample size.

### Accession number

The fastq files of all samples (bacterial 16S rRNA V1-V2 region sequence reads) were deposited in NCBI's Sequence Read Archive under accession number SRP058905.

## Results

### Hydrological characteristics of three meromictic lakes

Water profiles of Lakes Shira, Shunet and Oigon indicated a salinity-stratified distribution (Table 1). All three lakes had similar profiles in terms of temperature, pH, dissolved oxygen decrease and H_2_S, salinity, P, NH_4_^+^ and conductivity which increased with depth (Table 1). Oxic and anoxic layers of all lakes differed in several hydrological parameters; for example, salinity was lower in oxic (range, 14–28.42 g/L) than anoxic (18.84–81 g/L) layers of all three lakes. In addition, high concentrations of H_2_S were detected only in the stable anoxic layer of all lakes, being particularly high (range, 13.46–510 mg/L) in Lake Shunet. In Lake Shira, concentrations of NO_2_^-^ and NO_3_^-^ fluctuated across the two water layers and were not simple gradients, but these parameters increased from the oxic to anoxic layers in Lake Oigon. Finally, in Lake Shira and Lake Shunet, there was a distinct thermocline between the oxic and anoxic layers that was not detected in Lake Oigon.

**Table 1:** Physicochemical parameters of Lakes Shira, Shunet and Oigon.

Sample	Lake	Salinity	pH	t	k	D.O	H_2_S	P	NH_4_^+^	NO_2^-^_	NO_3^-^_
	m	g/L		°C	mS/cm	mg/L	mg/L	mg/L	mg/L	μg/L	μg/L
	Shira										
Oxic	SR1	14.00	8.62	19.80	16.66	8.61	0.00	0.00	0.05	9.94	49.89
	SR3	14.02	8.64	19.80	16.68	8.54	0.00	0.01	0.07	15.52	38.50
	SR5	16.69	8.53	12.08	19.90	9.61	0.00	0.02	0.01	0.80	1.50
	SR7	18.09	8.30	3.53	21.59	6.94	0.00	0.00	0.04	1.60	7.20
	SR9	18.56	8.29	1.64	22.17	5.55	0.00	0.00	0.05	2.20	10.00
	SR11	18.75	8.36	0.55	22.40	4.39	0.00	0.01	0.05	16.57	83.43
	SR12	18.79	8.36	-0.07	22.44	4.01	0.00	0.01	0.18	10.49	53.61
Anoxic	SR14	18.84	8.35	-0.01	22.51	0.38	0.00	0.01	0.02	1.30	52.9
	SR15	18.83	8.36	-0.20	22.49	0.00	1.71	0.03	0.03	0.19	63.84
	SR16	18.86	8.36	0.00	22.53	0.00	1.08	0.08	0.12	0.00	1.00
	SR17	18.78	8.33	0.10	22.42	0.00	2.99	0.36	0.50	2.01	33.07
	SR19	18.81	8.30	0.20	22.47	0.00	6.73	0.57	1.13	8.01	34.90
	SR21	18.79	8.24	0.48	22.45	0.00	16.50	1.23	3.39	11.61	42.60
	SR23	18.83	8.17	0.79	22.46	0.00	21.28	1.75	6.45	7.90	75.00
	Shunet										
Oxic	SN1	17.60	7.05	9.80	22.60	8.53	0.00	0.50	0.25	0.00	7.00
	SN2	17.60	7.05	11.16	22.60	8.37	0.00	2.00	0.00	0.00	7.00
	SN3	17.60	7.05	11.30	22.60	8.14	0.00	6.00	0.00	2.20	16.00
	SN4	23.70	6.95	6.50	28.12	8.22	0.00	23.00	0.97	2.60	10.00
Anoxic	SN5	33.50	7.10	1.30	36.95	0.00	13.46	-	-	-	-
	SN5.5	60.00	6.18	1.27	59.70	0.00	40.44	13.0	-	-	-
	SN6	81.00	5.87	1.27	81.40	0.00	510.00	-	-	-	-
	Oigon										
Oxic	OG0	24.76	9.02	18.57	34.06	6.53	0.00	0.12	0.62	0.00	21.50
	OG1	24.56	9.25	19.41	34.43	4.98	0.00	0.01	0.53	0.00	20.00
	OG2	24.54	9.23	19.32	34.33	4.75	0.00	0.00	0.51	0.10	19.40
	OG3	24.11	8.96	20.89	34.93	3.71	0.00	0.19	0.50	0.30	20.40
	OG4	24.09	8.95	20.81	34.85	2.95	0.00	0.00	0.36	0.23	20.70
	OG5	24.55	8.99	19.49	34.47	3.81	0.00	0.03	0.43	0.00	27.50
	OG6	24.73	9.02	18.30	33.81	2.76	0.00	0.10	0.46	0.00	24.90
	OG7	28.42	9.04	14.27	35.00	1.55	0.00	0.40	0.82	0.00	15.50
Anoxic	OG7.75	34.28	8.84	15.23	-	0.36	0.00	0.03	0.67	0.00	30.10
	OG8	29.09	9.10	12.28	33.81	0.12	3.0	0.42	2.73	0.10	25.70
	OG8.25	34.90	8.88	14.61	-	0.00	10.0	0.00	1.64	1.01	24.20
	OG8.5	33.76	8.90	13.99	34.10	0.00	25.0	0.00	1.33	1.09	23.00
	OG8.75	31.98	8.97	13.42	-	0.00	50.0	0.00	1.41	1.20	28.40
	OG8.85	32.00	8.90	14.00	-	0.00	50.0	0.00	1.40	1.10	29.00
	OG9	28.05	8.88	14.52	34.79	0.00	50.0	0.00	1.40	1.08	33.30

t-Temperature, k-Conductivity, D.O–Dissolved Oxygen

### Bacterial OTU richness and diversity

Rarefaction curves derived from the samples did not plateau, as OTU numbers continued to increase as more sequences were added (S3 Fig). Furthermore, anoxic samples had more OTU’s than the oxic (average of 191.5 versus 171, respectively). Bacterial diversity and richness in Lake Shunet were much greater than in Lakes Shira and Oigon (Table 2 and S3 Table). The greatest bacterial diversity between oxic and anoxic water layers was in Lake Oigon, with lowest diversity in oxic layers (Shannon 2.93, Simpson 0.15, and evenness 0.62) and the highest in anoxic layers (Shannon 4.93, Simpson 0.02, and evenness 0.87). There were no clear differences in any of Lake Shunet and Lake Shira’s bacterial community structure indicators (richness, diversity, and evenness) among the limnic water layers. However, OTU richness was greatest in Lake Shunet, intermediate in Lake Oigon and lowest in Lake Shira (Table 2).

**Table 2:** Estimated diversity indices and richness for the bacterial communities as represented in the 16S rRNA gene libraries^a^.

Sample	Lake	N^b^	S^c^	E^d^	R^e^	H^f^	Simpson	Chao1	Ace	Coverage
	Shira									
Oxic	SR1	831	119	0.79	16.44	3.79	0.04	181	216	0.94
	SR3	831	122	0.73	20.55	3.52	0.07	213	337	0.93
	SR5	831	113	0.74	18.84	3.50	0.06	204	280	0.93
	SR7	831	100	0.70	15.41	3.22	0.09	168	205	0.95
	SR9	831	157	0.77	30.14	3.91	0.04	321	545	0.89
	SR11	831	162	0.73	30.48	3.71	0.07	303	439	0.89
	SR12	831	180	0.79	33.22	4.13	0.04	348	496	0.88
Anoxic	SR14	831	147	0.76	26.03	3.80	0.05	264	350	0.91
	SR15	831	139	0.76	22.61	3.74	0.06	241	309	0.92
	SR16	831	155	0.78	28.09	3.94	0.04	304	434	0.90
	SR17	831	124	0.75	20.89	3.63	0.06	227	313	0.93
	SR19	831	110	0.74	18.84	3.46	0.06	195	291	0.93
	SR21	831	161	0.80	27.74	4.07	0.04	294	409	0.90
	SR23	831	117	0.66	20.55	3.15	0.13	215	318	0.93
	Shunet									
Oxic	SN1	831	263	0.84	55.14	4.67	0.03	617	927	0.81
	SN2	831	304	0.89	65.08	5.06	0.01	740	1190	0.77
	SN3	831	285	0.86	60.97	4.88	0.02	684	1077	0.79
	SN4	831	259	0.86	53.09	4.78	0.02	581	918	0.81
Anoxic	SN5.0	831	283	0.86	60.28	4.84	0.02	660	1024	0.79
	SN5.5	831	254	0.80	56.86	4.42	0.04	646	1078	0.80
	SN6	831	242	0.80	53.43	4.40	0.04	586	1030	0.81
	Oigon									
Oxic	OG0	831	154	0.78	29.11	3.93	0.04	322	521	0.90
	OG1	831	187	0.74	34.59	3.89	0.08	334	496	0.88
	OG2	831	150	0.71	27.74	3.56	0.07	268	404	0.90
	OG3	831	157	0.71	28.09	3.60	0.11	296	408	0.90
	OG4	831	112	0.62	22.26	2.93	0.15	249	486	0.92
	OG5	831	153	0.73	27.74	3.65	0.07	289	428	0.90
	OG6	831	190	0.79	37.68	4.16	0.04	413	651	0.87
	OG7	831	201	0.77	39.39	4.10	0.05	424	616	0.86
Anoxic	OG7.75	831	204	0.80	42.13	4.26	0.03	457	803	0.85
	OG8.00^*g*^	831	191	0.80	34.59	4.21	0.03	334	484	0.88
	OG8.25	831	166	0.78	32.20	3.97	0.04	370	462	0.89
	OG8.50	831	246	0.83	47.61	4.56	0.03	459	673	0.83
	OG8.75	831	187	0.78	36.65	4.11	0.04	373	622	0.87
	OG8.85	831	284	0.87	56.51	4.93	0.02	571	850	0.80
	OG9	831	245	0.84	49.66	4.60	0.02	499	842	0.82

^*a*^Calculations were based on OTUs formed at evolutionary distance of <0.03(or~97% similarity).

^*b*^*N* = the number of sequences.

^*c*^*S* = the number of OTUs.

^*d*^Evenness = Shannon/In(the number of OTUs)

^*e*^Richness = (number of singleton OTUs-1)/log_10_N. The maximum value is (N-1)/log_10_N.

^*f*^*H =* Shannon diversity index

^*g*^Equal sampling power (OG8.00-lowest read numbers 831) applied to all samples.

### Bacterial community composition in oxic and anoxic layers

**Fig 2:**
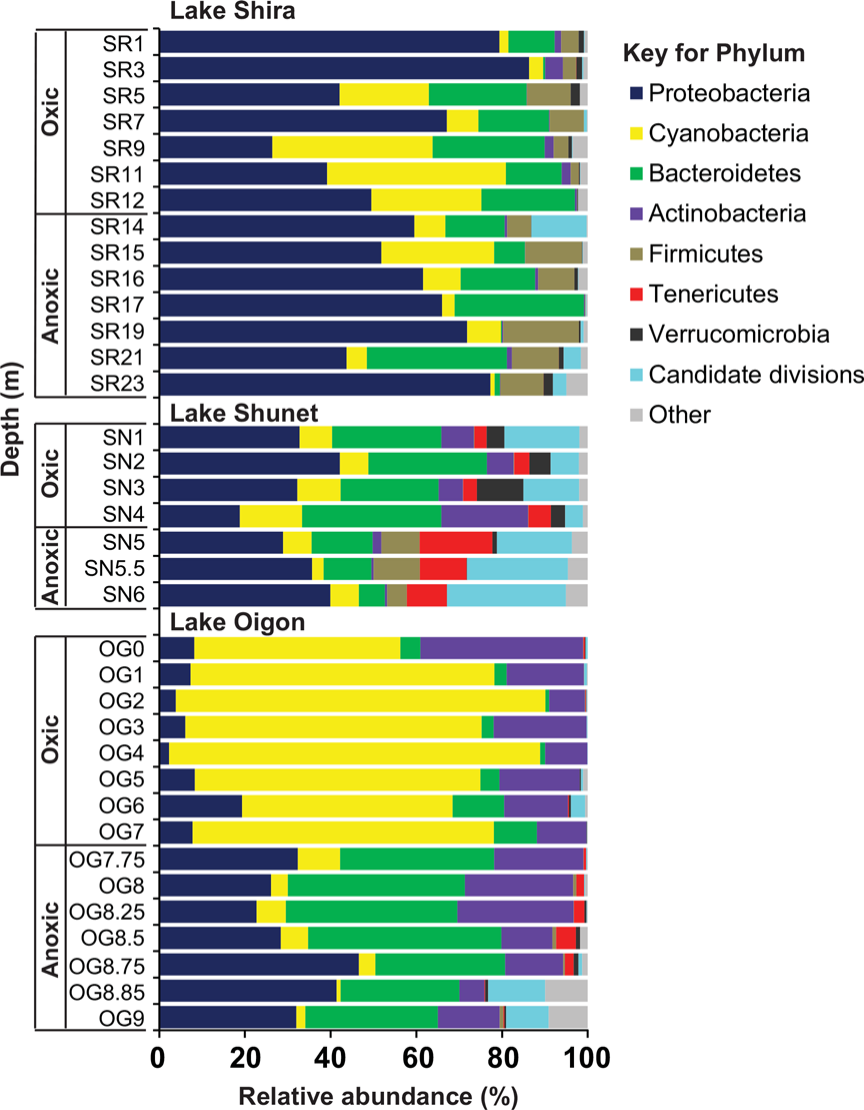
Relative abundance of bacterial communities at the phylum level according to depth in Lakes Shira, Shunet and Oigon.

A total of 120,510 qualified sequences were retained (average sequence length of 300 bp) and clustered into 6672 OTUs. All qualified sequences were classified below the domain level of bacteria. Bacterial community members consisted of 42 phyla (23 established phyla and 19 candidate phyla), 97 classes and 665 genera. The relative abundance of bacterial communities at various taxonomic levels was determined. At the phylum level (Fig 2), *Proteobacteria*, *Cyanobacteria*, *Bacteroidetes* and *Actinobacteria* were dominant groups in oxic and anoxic layers of all the three lakes; in that regard, these four phyla comprised > 60% of relative abundance in each layer. *Proteobacteria* was the dominant group in the anoxic layer of Lake Shira (~64% of relative abundance), Lake Shunet (~43.2%) and Lake Oigon (~33.8%), respectively. *Cyanobacteria* were mainly detected in oxic layer of all lakes, particularly high in the oxic layer (~68.2% of relative abundance) of Lake Oigon. *Actinobacteria* was the major bacterial phylum in oxic (~17.4% of relative abundance) and anoxic (~17.9%) layers of Lake Oigon, but was only a minor group in the other two lakes. Many of the dominant phyla were not detected in specific lakes. For example, *Firmicutes* was a major group in both layers of Lake Shira and anoxic layer of Lake Shunet, but quite rare (~0.1% of relative abundance) in Lake Oigon. *Tenericutes* was a large proportion of bacterial community in both layers of Lake Shunet and anoxic layer of Lake Oigon, but not detected in Lake Shira (Fig 2).

**Fig 3:**
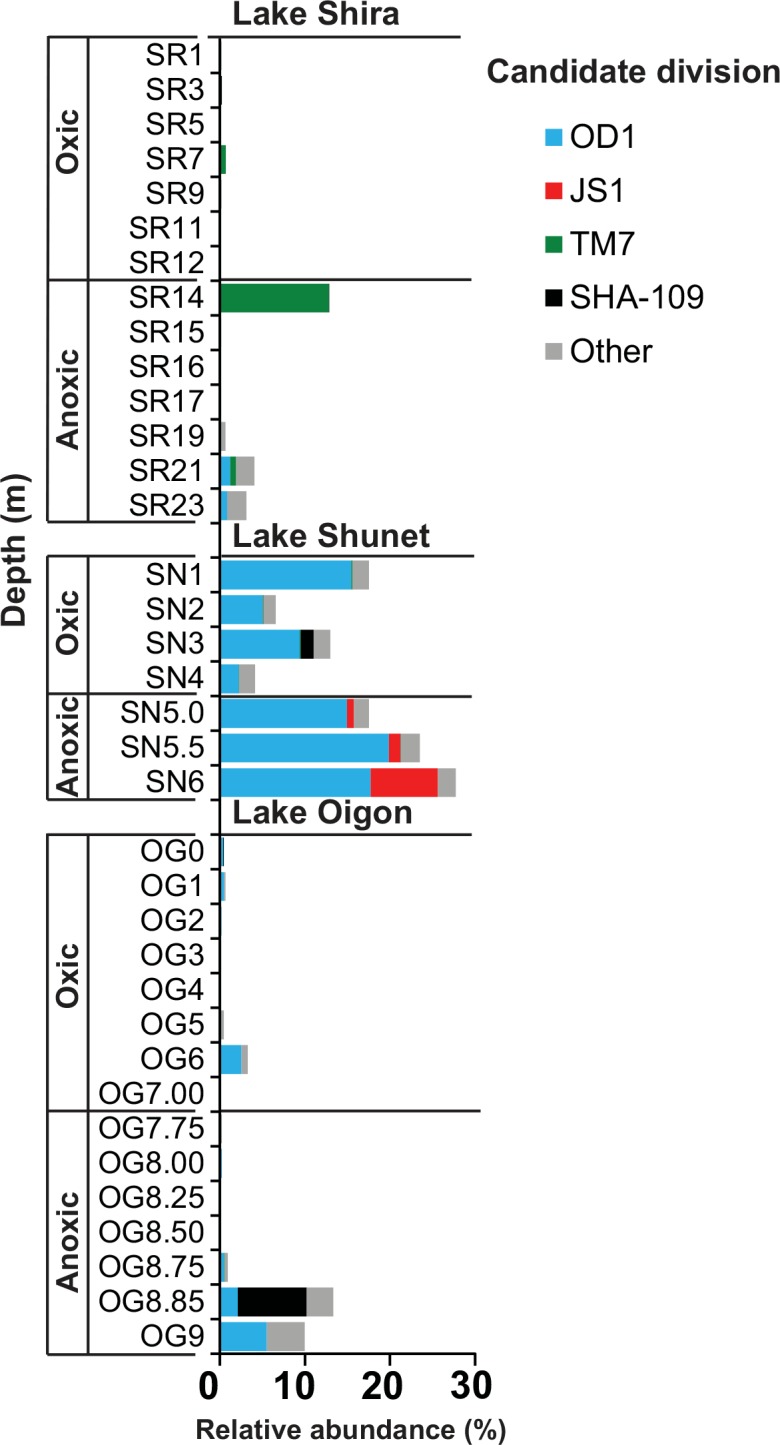
Bar charts representing distribution and relative abundance of bacterial candidate division among the two main limnetic layers in Lakes Shira, Shunet and Oigon. The size of the bar indicates relative abundance.

Many candidate divisions (phylum level) were detected in these three lakes. In taxonomic classification using SILVA ngs, substantial percentages (3.94%) of sequences from the lakes were classified into 19 candidate divisions, especially division OD1, the highest percentage (23.12%) in anoxic layer of Lake Shunet (Fig 3). Furthermore, only division OD1 was dominant along stratification zones in Lake Shunet. Based on sequence similarity, a phylogenetic tree was constructed using OD1-related sequences (S4 Fig). There were clear differences between oxic and anoxic layers, with all individual OTU’s being major components at only one particular layer in Lake Shunet. Therefore, various OD1 species specifically distributed between oxic and anoxic layers of Lake Shunet, that is, anoxic and oxic OD1 related sequences, were clustered into two disparate groups (S4 Fig). In addition, most candidate phyla dominated at particular layers, such as JS1, SHA-109 and TM7, which were only detected in anoxic layers. Percentages of candidate divisions were higher in the anoxic layer than in the oxic layer of the three lakes (paired Wilcoxon’s test, *P* < 0.028).

**Fig 4:**
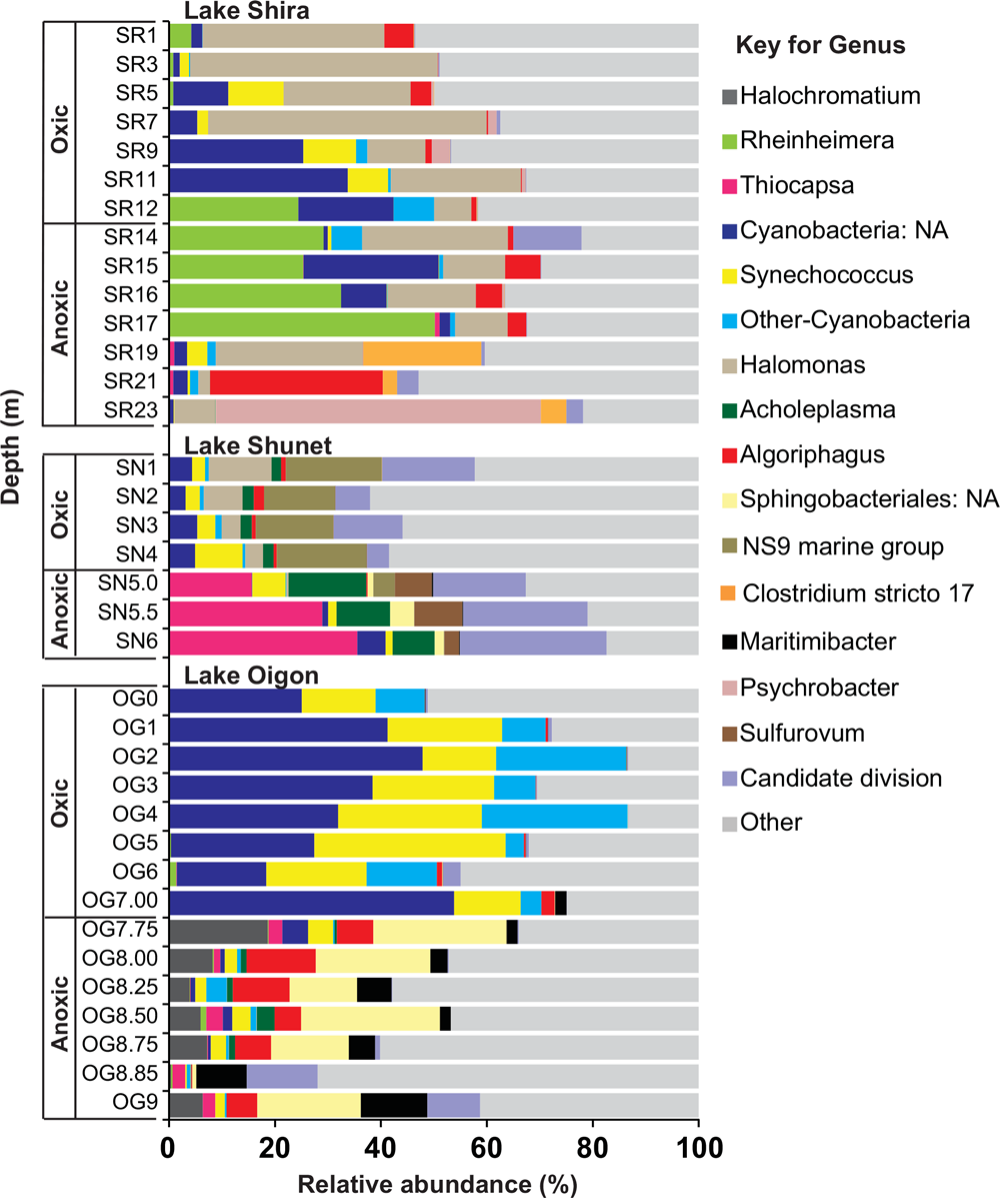
Relative abundance (genus level) of bacterial communities along depth in Lakes Shira, Shunet and Oigon.

Relative abundances and distributions of bacterial communities (genus level) across the main water layers were visualized in a bar plot (Fig 4). Bacterial communities were specific for individual lakes. For example, three closely related genera of PSB (*Rheinheimera*, *Thiocapsa* and *Halochromatium)* were predominant bacterial components in the anoxic layer of Lake Shira (~20.6% of relative abundance), Lake Shunet (~27.1%) and Lake Oigon (~9.25%), respectively (Fig 4). However, only *Halomonas* was commonly detected along both stratification layers in Lake Shira. Most groups were predominant (or only detected) within particular layers. For example, NS9 marine group was detected in the oxic layer, whereas *Sulfurovum*, *Algoriphagus* and an unclassified group of *Sphingobacteriales* were detected only in the anoxic layer (Fig 4). Furthermore, *Cyanobacteria* were also much more abundant in oxic layers of all lakes, representing almost 50% of all oxic-related genera in Lake Oigon.

Transition layers could be crucial in species compositional distribution of PSB among meromictic lakes (S5 Fig). Since some PSB were not only detected in the anoxic layer, but also in the oxic layer, their phylogenetic lineages were further examined. Clearly, there were two broadly classified groups between the oxic and anoxic layers (S5 Fig).

### Comparison of bacterial communities in the meromictic lakes

**Fig 5:**
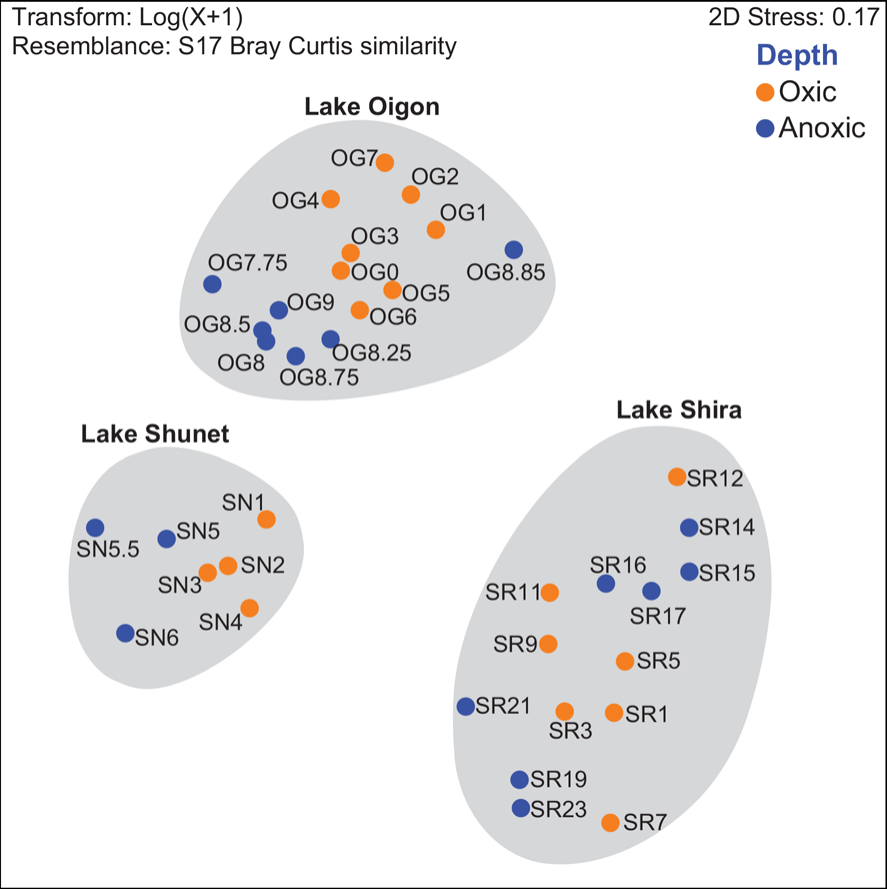
Non-metric multidimensional scaling (NMDS) analysis (Bray–Curtis similarity matrix) based on relative abundance of bacteria along the stratified water column in three lakes. Orange and blue indicate oxic and anoxic layers, respectively.

Both statistical and non-statistical methods (i.e., nMDS and clustering/heatmap, respectively) were used to compare similarities among bacterial communities among the three lakes. Based on nMDS plotting, there were clear differences among lakes, as well as significant variation between oxic and anoxic layers within each lake (Fig 5). Bacterial community composition had nearly perfect separation of its two layers in Lake Shunet (as visualized in the nMDS plot). However, bacterial communities in the anoxic layer of Lake Shira were not clearly similar to each other, consistent with bar charts (Fig 4) of its bacterial community structure.

**Fig 6:**
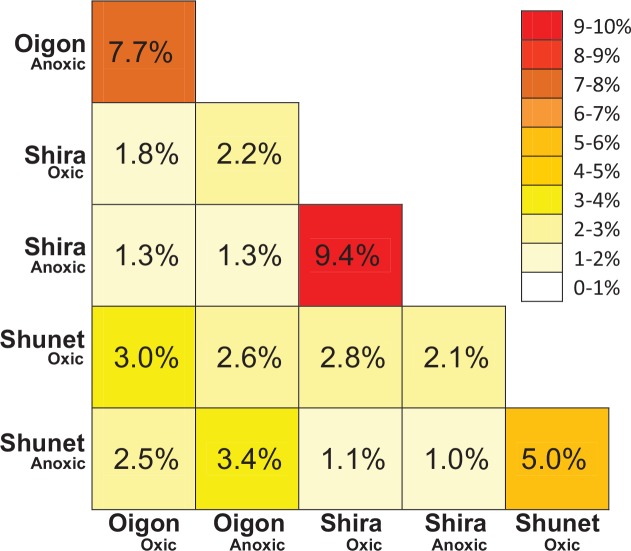
Number of shared OTUs in Lakes Shira, Shunet and Oigon, visualized by a heat map. Relative abundance of OTUs were standardized to the total number of OTUs (per-lake basis).

In addition, distributions of the shared OTUs along the vertical gradient in the three lakes were visualized with a clustering-heat map to test the relationship between each sampled layer (Fig 6). Bacterial communities in oxic layers of the lakes differed (*P*<0.05) from those in anoxic layers (only 5.0–9.4% similar) according to a heat-map ordination plot (Fig 6). Although most of the OTUs (range 90.6–95%) appeared only at particular layers (that is, oxic or anoxic) in each lake (Fig 6), bacterial compositions between layers within individual lakes were more similar to each other than those between two lakes.

### Relationship between hydrological parameters and bacterial community structure

**Fig 7:**
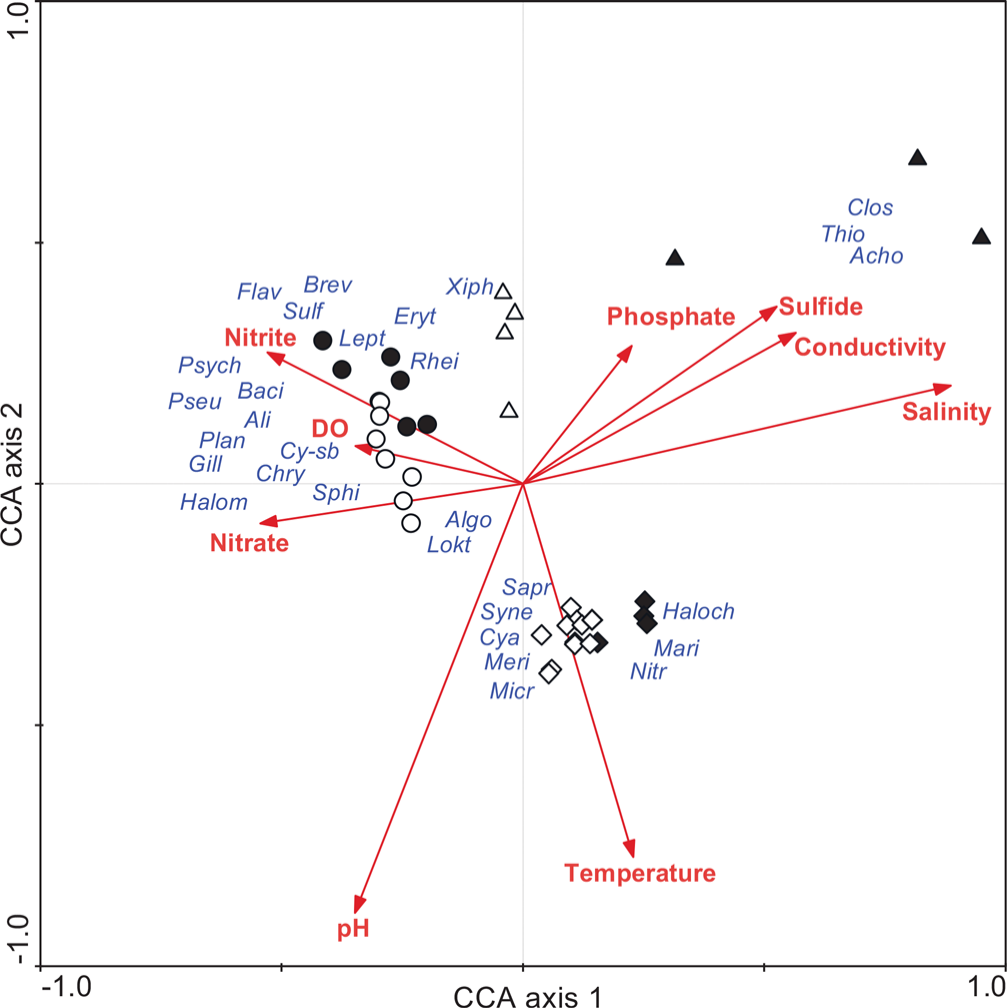
Canonical correspondence analysis (CCA) of Lake Shira (●Anoxic, o Oxic), Lake Shunet (▲Anoxic, △ Oxic), and Lake Oigon (◆ Anoxic, ◇Oxic) combined, based on abundant taxa of bacteria (>0.1% of the total community). Environmental variables that significantly influenced the bacterial community are represented as vectors; the length of the arrow corresponds to the degree of significance. The genus name is abbreviated as follows. **(***Thio-Thiocapsa*, *Rhei-Rheinheimera*, *Haloch-Halochromatium*, *Halom-Halomonas*, *Cy-sb-Cyanobacteria*.*sp*, *Cya-Cyanobacteria-FamilyI*, *Syne-Synechococcus*, *Acho-Acholeplasma*, *Algo-Algoriphagus*, *Pseu-Pseudomonas*, *Psych-Psychrobacter*, *Flav-Flavobacterium*, *Lokt-Loktanella*, *Nitr-Nitriliruptor*, *Xiph-Xiphinematobacter*, *Brev-Brevundimonas*, *Plan-Planococcus*, *Gill-Gillisia*, *Eryt-Erythrobacter*, *Meri-Merismopedia*, *Chry-Chryseobacterium*, *Sulf-Sulfurovum*, *Lept-Leptolyngbya*, *Baci-Bacillus*, *Sphi-Sphingomonas*, *Sapr-Saprospiraceae-uncultured*, *Mari-Maritimibacter*, *Micr-Microbacteriaceae-uncultured*, *Ali-Aliidiomarina*, *Clos-Clostridium sensu stricto 17)*.

The CCA analysis was done to determine relationships between environmental variables per sampling layer of the three lakes, as well as associations between abundant bacterial genera (>0.1% of the total community) and physico-chemical parameters. Based on this approach, bacterial communities of the lakes were significantly correlated with temperature, pH, nitrate, nitrite, phosphate, conductivity, dissolved oxygen, H_2_S and salinity (*P* < 0.05; Fig 7). Furthermore, some specific bacterial groups correlated closely with particular environmental variables on the CCA plot; for example, *Sulfurovum* was associated with H_2_S, phosphate, and NH_4_^+^, whereas *Psychrobacter* and *Planococcus* were associated with salinity. Moreover, CCA analysis also indicated that bacterial communities in anoxic layers were more closely associated (*P*<0.05) with conductivity, sulfide, phosphate and salinity (Fig 7). In that regard, bacterial community structures in the oxic layers of the three lakes were correlated with temperature, pH and dissolved oxygen (*P*<0.05; Fig 7).

## Discussion

This study was a detailed characterization of bacterial diversity and community composition in three high-latitude meromictic lakes near central Asia. Based on high variations and uniqueness of bacterial community in the three lakes, we concluded that meromictic ecosystems were characterized by high diversity and heterogeneity of niches.

### Bacterial diversities are high in the anoxic layer

Bacterial diversity in the anoxic layer of Lake Oigon was high in this study, with Shannon-Weaver values up to 4.93, which was apparently higher than the 4.36 in Lake Mahoney [18], 3.6 in Lake Pavin [24], 1.8 in Lake Nyos [19], 1.01 in Organic Lake [47], up to 2.5 in lakes of the Monegros Desert [48] and 3.83 in Lake Soap [49]. To the best of our knowledge, this was among the greatest diversity ever reported in a meromictic lake bacterial community. However, we cannot exclude potential bias due to methodology. In that regard, the extreme diversity could have been due, at least in part, to the use of massively parallel pyro-sequencing (although it is a more effective and sensitive approach for detecting bacterial diversity compared to traditional approaches [50]).

Why was bacterial diversity consistently greater in the anoxic versus oxic layer? This phenomenon has been reported in Lake Mahoney [18], Ace Lake [14], Ursu Lake and Fara Fund Lake [8]. Perhaps abundant nutrients in the anoxic layer promote diversity. Furthermore, it has been proposed that the mineralization process (organic compounds becoming impregnated by inorganic compounds in anoxic systems) was a multistep process that needed a diverse and complex bacterial community [14, 51]. Additional explanations for higher diversity in the anoxic layer might be related to a relatively lack of mixing (*i*.*e*., homogenization) in the anoxic layer and downward metabolic fluxes [3, 8, 29, 34].

### Bacterial communities are specific between oxic and anoxic layers

In all lakes, bacterial community and composition results had clear vertical separation between oxic and anoxic layers. These two layers hosted distinctly different bacterial taxa (Fig 5), with only a few shared OTU’s (Fig 6). Similarly, previous meromictic lake studies also identified disparate bacterial communities in the oxic versus anoxic layers [4, 8, 14, 18, 52]. Vertical distributions of bacterial communities in Lakes Shira, Shunet and Oigon were generally similar to those of other meromictic lakes in terms of trophic characteristics [10, 20, 32]. However, variations in internal environmental factors may be the primary explanation for diversification of bacterial community structures. Previous studies also suggested that environmental gradients had a role in separating oxic bacterial taxa from anoxic taxa, because oxygen-sensitive sulfur and nitrogen cycles occurred in the anoxic layer [3, 8, 18, 50]. Additional studies provided more evidence [13, 50] to support the finding that observed bacterial groups were significantly associated with variation along the water column, although other factors (e.g. anthropogenic effects) cannot be excluded.

In addition, local bacterial occupants further diversified local environmental characters interactively; ultimately each lake was a unique variation on the meromictic lake theme [13, 16, 53]. Distinct bacterial community compositions in the two layers were attributed to different mixing regimes, as well as disparate physicochemical milieus [8, 20, 54, 55]. Therefore, this may also explain why bacterial communities and physicochemical characteristics frequently differ between oxic and anoxic layers of meromictic lakes, regardless of their similarities in lake profile.

### Purple sulfur bacteria are taxonomically variable among meromictic lakes

Anoxygenic phototrophic bacteria are hallmark microorganisms of meromictic lakes. In that regard, PSB and GSB are the two most representative members in the anoxic layers [6, 7, 10, 56, 57]. Three discrete dominant PSB were identified in the three lakes. That species most closely related to *Halochromatium roseum* dominated in Lake Oigon was a new discovery, distinct from the other two lakes. In Lake Shunet, there was a high relative abundance of species most closely related to *Thiocapsa rosea*, which supported previous observations [10, 29, 33]. However, the dominant PSB, *Rheinheimera*-like genus in Lake Shira differed from a PSB, *Lamprocystis purpurea*, in a previous report [9, 32], which identified the species based on morphology and pigment composition. Species-specific detection of PSB communities in these lakes was done using culture-dependent approaches, which may have made it difficult to identify dominant PSB species. For example, in this study, more than 100 OTU’s related to PSB were detected in Lake Shira. These PSB species had similar physiological features, e.g. color of cell suspension, aggregate formation, cell size, pH and temperature ranges for growth, formation of gas vesicles, and lack of salt requirement [58].

However, that they have disparate genetic backgrounds clearly justified the maintaining of several discrete genera in a phylogenetic orientated taxonomy.

Most PSB are obligate or facultative photoautotrophs, but *Rheinheimera* spp, a group of PSB, are chemoorganotrophes [59]. Many *Rheinheimera*-like sequences were detected in Lake Shira; however, our identified *Rheinheimera*-like sequences were not clear, because sequence identity was only ~90% similar at their family level of taxonomic thresholds of bacteria [60], suggesting the dominant PSB in Lake Shira was indeed a novel type of bacteria.

Based on the present and previous reports, we inferred that dominant PSB genera in meromictic lakes were highly variable, as shown by *Thiocapsa* in Lake Shunet, a novel genus in Lake Shira, *Halochromatium* in Lake Oigon, *Lamprocystis purpurea* in Lake Mahoney [18], and *Thiodictyon syntrophicum* in Lake Cadagno [61]. In the present study, although Lake Shira and Lake Shunet were in close proximity (8 km), dominant PSB species clearly differed between the two lakes. Similar results have been described [15, 29, 32]. For example, dominant species of phototrophic sulfur bacteria differed between Lake Ciso and Lake Vilar, even though those two lakes are only 1 km apart [62]. However, why were no similar dominant PSB species detected among those lakes? Although the causes could be complex, perhaps the uniqueness and consistency of environmental profiles of each meromictic lake could reduce the probability that other foreign PSB species successfully colonized the lake.

### Bacterial communities in Lake Oigon

This study was the first bacterial community survey of Mongolian lake using a culture-independent method. The main difference in bacterial groups between Lake Oigon and other two meromictic lakes was *Actinobacteria* (mostly the genus *Nitriliruptor*). In that regard, this bacterial group was predominant in Lake Oigon (~17.7% of relative abundance), but was only a minor group in Lake Shunet (~5.6%) and Lake Shira (~1.0%). Members of the *Actinobacteria* are typically known as inhabitants of soil environments and freshwater [63]. However, based on this study and other reports, bacteria can also thrive in salt meromictic lakes [14, 63, 64]. The dominant *Nitriliruptor* was recovered from an alkaline soda lake and can survive alkaline water pH (~10) with optimum growth at pH 9.0–9.5 and moderate salt-tolerance [63]. That Lake Oigon was more alkaline than the other two lakes was consistent with more *Nitriliruptor* in Lake Oigon.

### Candidate phylum dominated in the anoxic layer of Lake Shunet

One of the most striking phenomena in this study was the large proportion of unclassified bacterial groups and candidate divisions (UBG/CD) detected in these meromictic lakes (Fig 3), particularly high in the anoxic layer (~23.1% relative abundance) of Lake Shunet. To our best knowledge, this was the greatest relative abundance of UBG/CD bacteria reported in meromictic lakes. In previous reports from lakes, there were also great proportions of UBG/CD at the sulfide-rich and anoxic layers. For instance, the proportion of bacteria that were UBG/CD was ~10% for Lake Mahoney [18], 7.37% for Lake Alinen Mustajarvi [15], and15% for Lake A [4]. It was noteworthy that UBG/CD may encounter extreme conditions in various ecological niches [8, 24, 64]. Furthermore, they have mainly been regarded as a dominant group in sulfur-rich and anoxic environments [15, 52, 65]. Perhaps the presence of many UBG/CD bacteria in the anoxic layer of meromictic lakes was due to extreme environmental conditions, including a high concentration of salt and hydrogen sulfide (Table 1). In that regard, that the anoxic layer of Lake Shunet was more extreme than other two lakes might account for the greater number of UBG/CD bacteria. In addition, that more bacteria in the anoxic layer of meromictic lakes were poorly described could be due, in part, to difficulties in anaerobic versus aerobic culture methods. Consequently, fewer anaerobic bacterial species have been characterized.

In conclusion, this study provided a high-resolution characterization of bacterial diversity and community in three meromictic lakes. Based on comparative analysis of bacterial composition, Lake Shira, Lake Shunet and Lake Oigon clearly had high diversity and uniqueness of bacterial communities between water layers within individual lake and among lake. Several hydrological parameters were significantly correlated with variations in the dominant bacterial groups along the water column; therefore, we speculated that there was an intimate relationship between microenvironments and specific bacterial communities. In addition, abundant unclassified species present in these lakes, particularly Lake Shunet, was evidence that meromictic lakes were hot spots for exploring bacterial diversity, taxonomy and phylogenetics. This study should serve as an initial inventory survey of the bacterial community and diversity of meromictic lakes in central Asia and provide an impetus for further studies, e.g. functional ecology and metagenomics to examine interactions between the bacterial community structure and environmental conditions in meromictic lakes.

## Supporting Information

S1 Fig The mechanism of the vacuum pump. (PDF)

S2 Fig Illustration of change in the read numbers at various steps of data processing. (PDF)

S3 Fig Rarefaction curves generated by number of sequences versus number of OTUs in Lakes Shira, Shunet and Oigon. (PDF)

S4 Fig A phylogenetic tree and relative abundance of OTUs affiliated with Candidate division OD1 in Lake Shunet. (PDF)

S5 Fig Distribution of dominant purple sulfur bacterial communities at the OTU (97% similarity) level in Lakes Shira, Shunet and Oigon along the vertically stratified water column visualized by a heat map. Sequence reads were with standardized total relative abundance of individual layers of these lakes.
(PDF)

S1 Table Sampling depths of Lakes Shira, Shunet and Oigon. (DOCX)

S2 Table Barcoded primers for obtaining the 16S rRNA amplifications from Lakes Shira, Shunet and Oigon water samples. The barcode was added at the 5’-end of the bacterial universal forward primer 27F and reverse primer 341R.
(DOCX)

S3 Table Estimated diversity indices and richness for the bacterial communities as represented in 16S rRNA gene libraries^*a*^. (DOCX)
