# Supplementary figures and images for: Bacterial Communities of Three Saline Meromictic Lakes in Central Asia
Source: PLoS One. 2016 Mar 2;11(3):e0150847. doi: 10.1371/journal.pone.0150847 (PMC4775032; doi:10.1371/journal.pone.0150847)

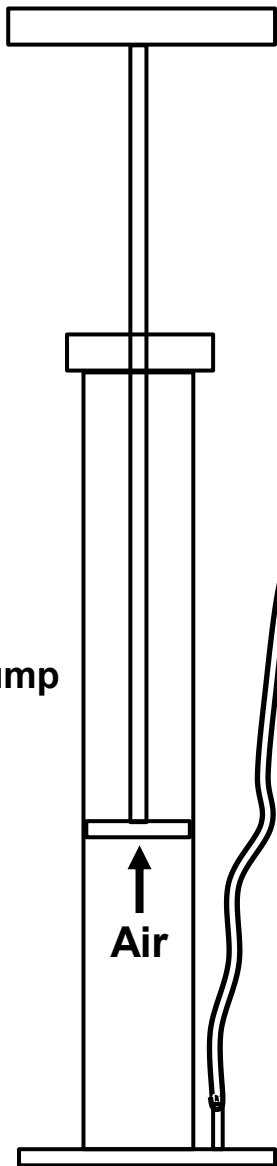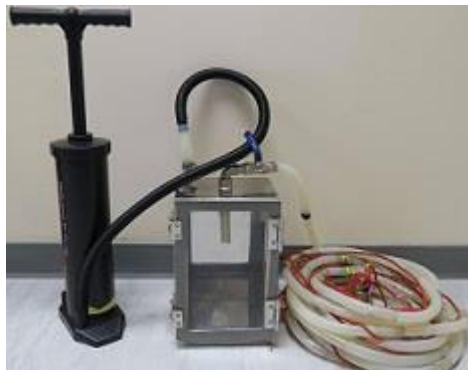

**Vacuum box**

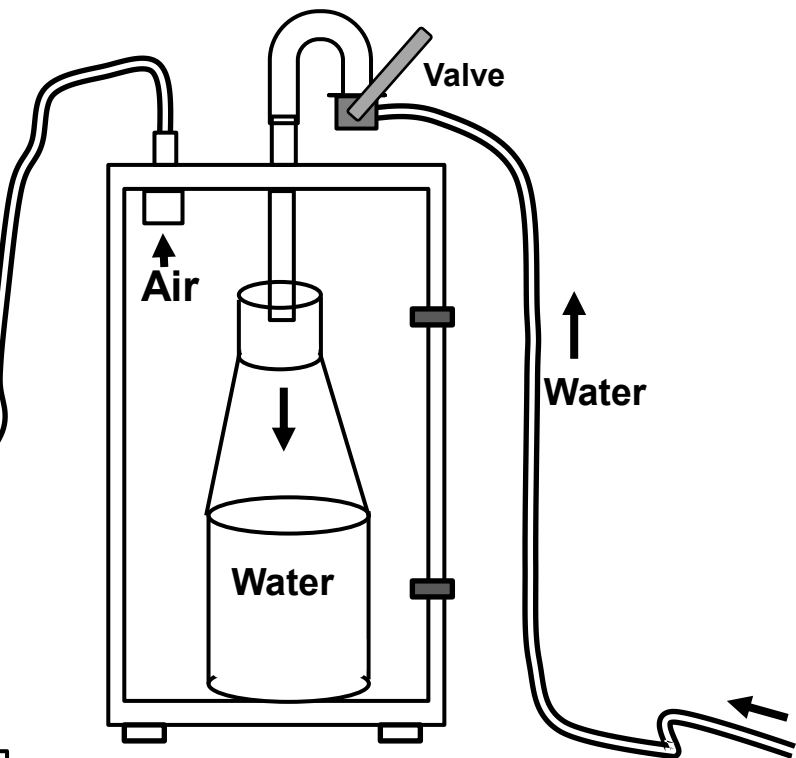

Supplement: S1 Fig — (PDF) [file pone.0150847.s001.pdf]

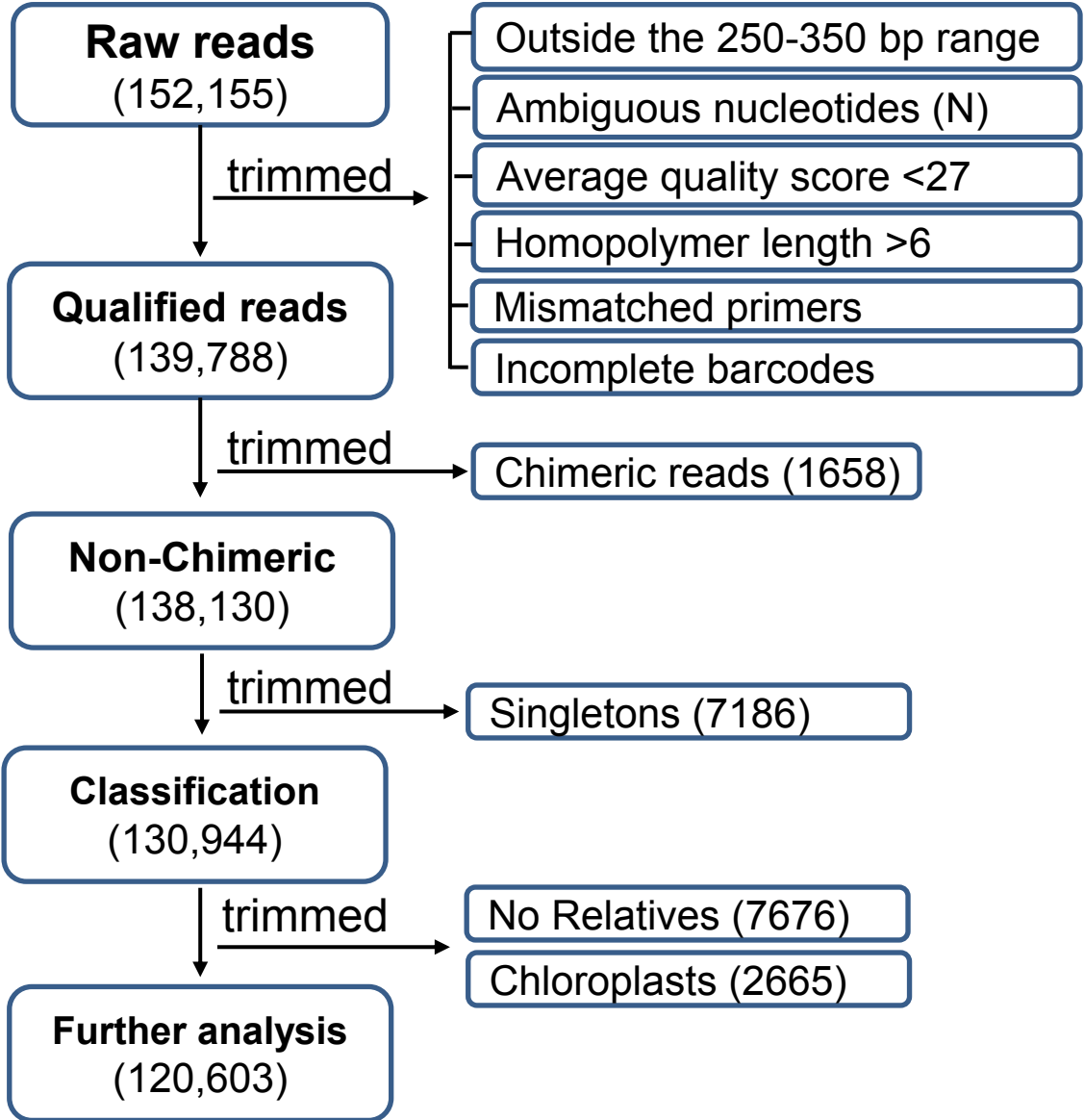

Supplement: S2 Fig — (PDF) [file pone.0150847.s002.pdf]

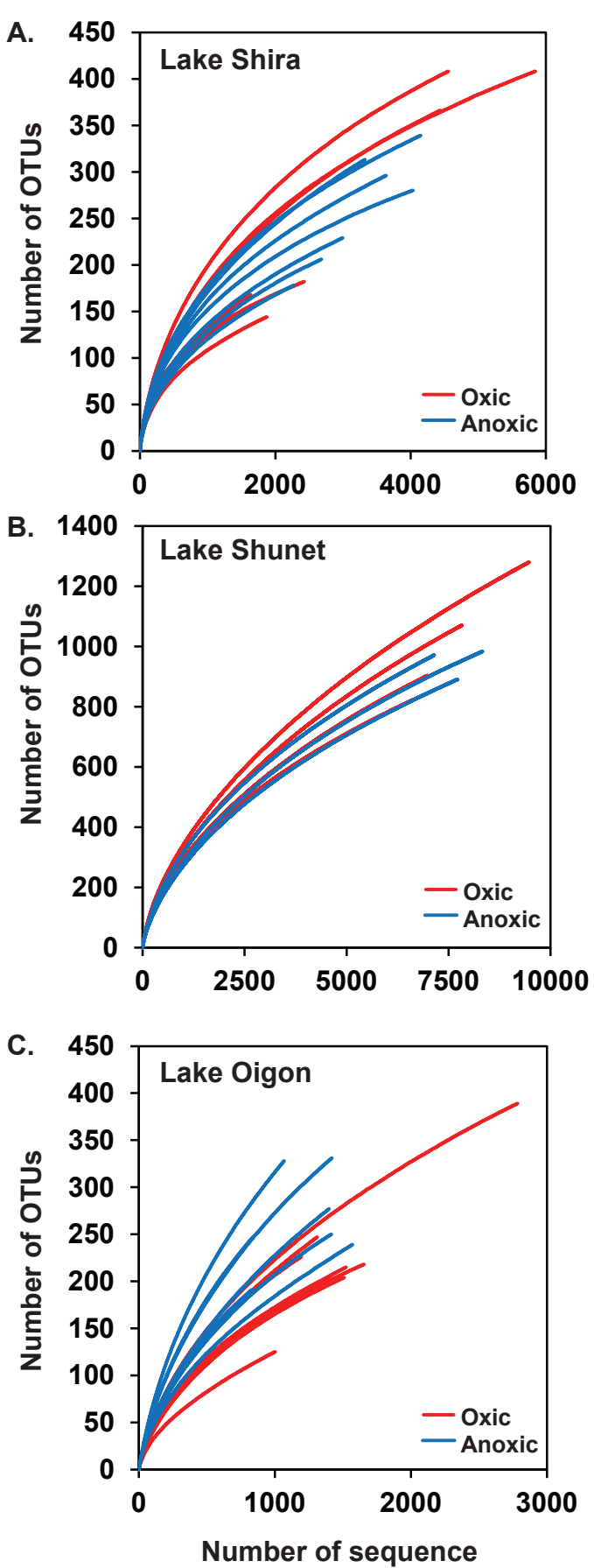

Supplement: S3 Fig — (PDF) [file pone.0150847.s003.pdf]

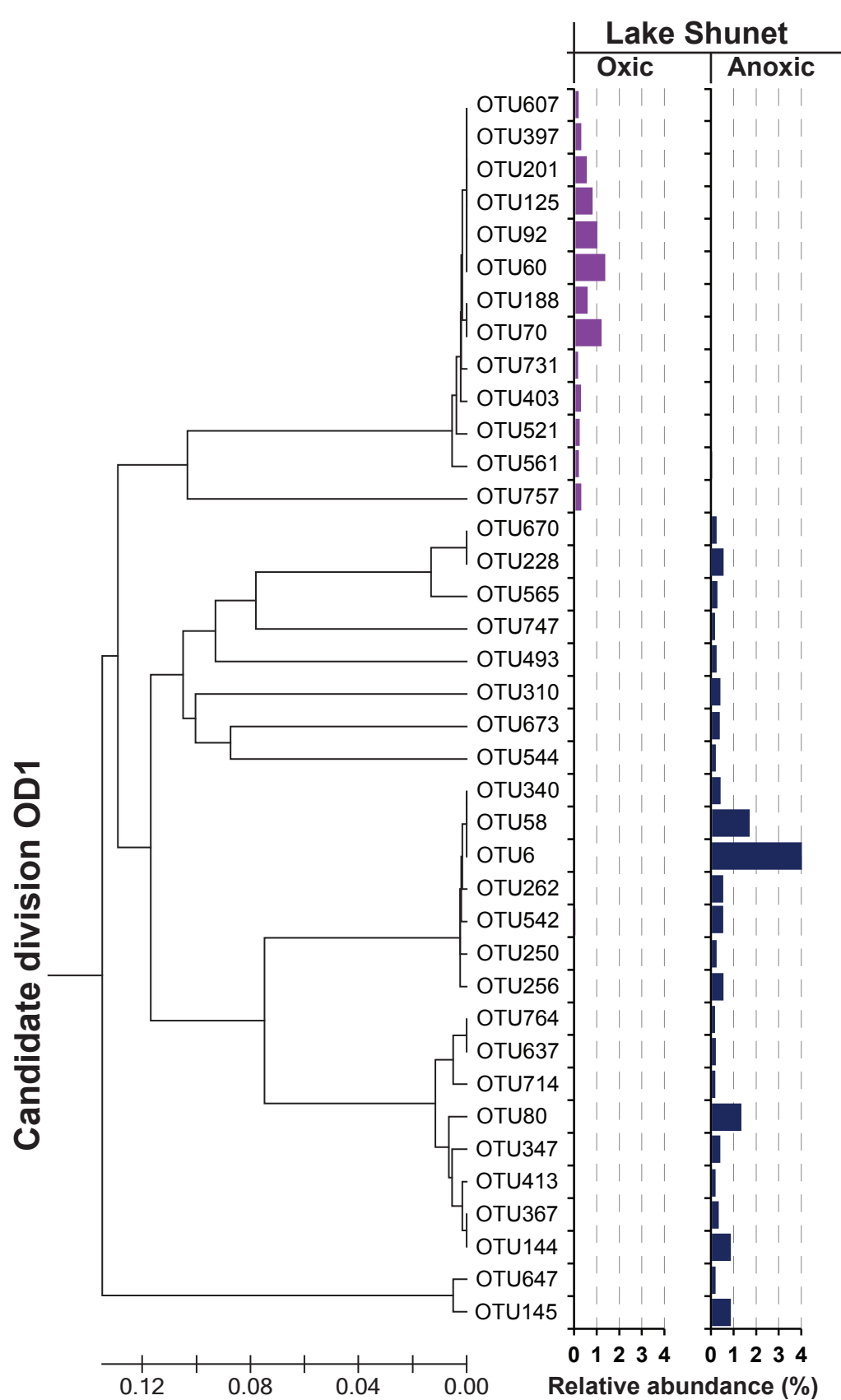

Supplement: S4 Fig — (PDF) [file pone.0150847.s004.pdf]

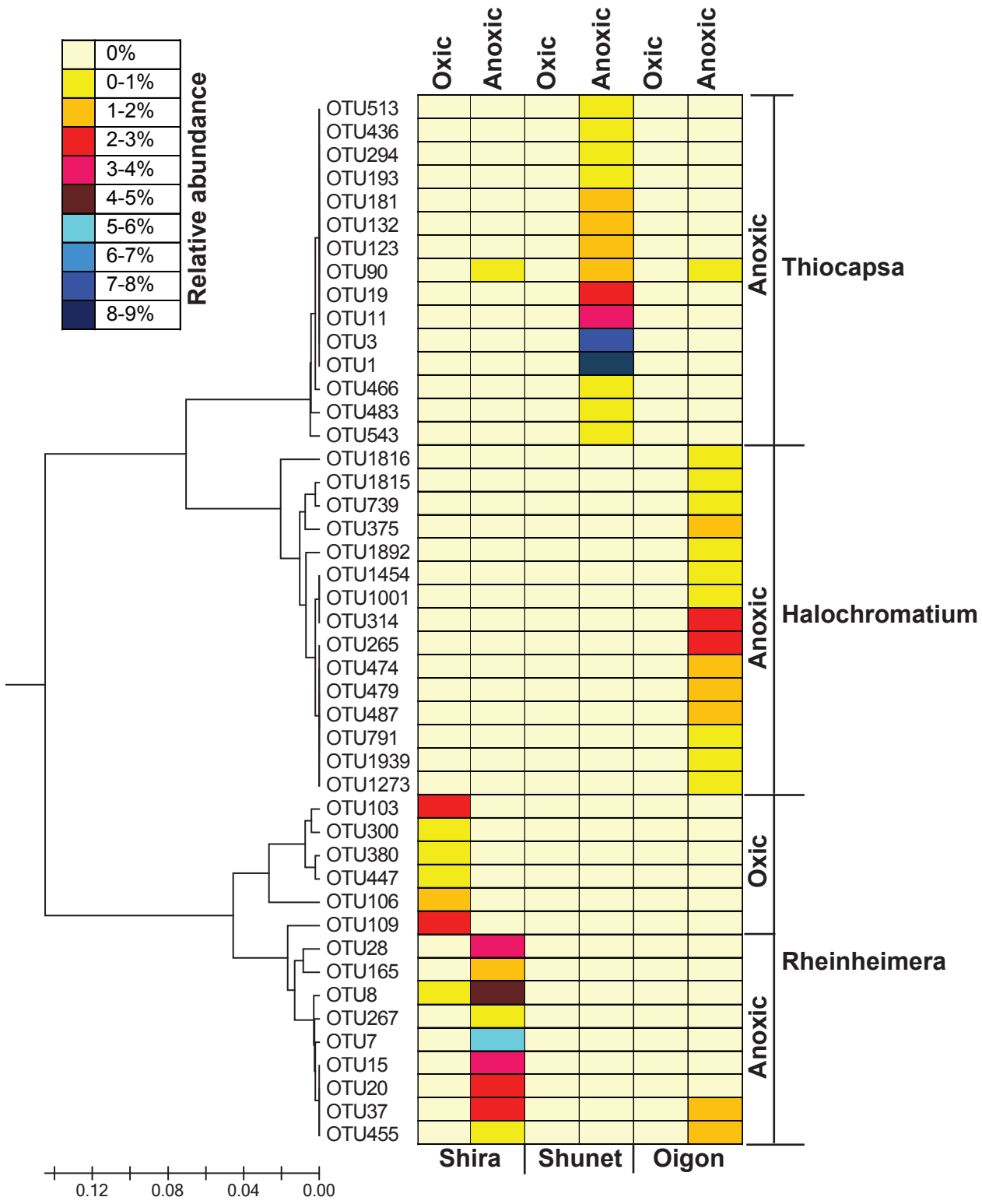

Supplement: S5 Fig — Sequence reads were with standardized total relative abundance of individual layers of these lakes. (PDF) [file pone.0150847.s005.pdf]
