# Supplementary material for: Bacterial Communities of Three Saline Meromictic Lakes in Central Asia
Source: PLoS One. 2016 Mar 2;11(3):e0150847. doi: 10.1371/journal.pone.0150847 (PMC4775032; doi:10.1371/journal.pone.0150847)
Supplement: S1 Table — (DOCX) [file pone.0150847.s006.docx]

**S1 Table.** Sampling depths of Lakes Shira, Shunet and Oigon

| Compartment | | **Lake Shunet** | **Lake Shira** | **Lake Oigon** |
| --- | --- | --- | --- | --- |
|  |  | Depth (m) | Depth (m) | Depth (m) |
| **Oxic** | **Mixolimnion** | 1  2  3  4 | 1  3  5  7  9  11  12 | 0  1  2  3  4  5  6  7 |
| **Anoxic** | **Chemocline** | 5  5.5  6 | 14  15  16  17  19  21  23 | 7.75  8  8.25  8.5  8.75  8.85  9 |
|  | **Monimolimnion** |  |  |  |
