# Supplementary material for: Bacterial Communities of Three Saline Meromictic Lakes in Central Asia
Source: PLoS One. 2016 Mar 2;11(3):e0150847. doi: 10.1371/journal.pone.0150847 (PMC4775032; doi:10.1371/journal.pone.0150847)
Supplement: S2 Table — The barcode was added at the 5’-end of the bacterial universal forward primer 27F and reverse primer 341R. (DOCX) [file pone.0150847.s007.docx]

**S2 Table.** Barcoded primers for obtaining the 16S rRNA amplifications from Lakes Shira, Shunet and Oigon water samples. The barcode was added at the 5’-end of the bacterial universal forward primer 27F and reverse primer 341R.

|  | **ID** | | **Barcoded forward primer 27F** | | **Barcoded reverse primer 341R** |
| --- | --- | --- | --- | --- | --- |
|  | | SA1 | | AACCAGAGTTTGATCMTGGCTCAG | AACCCTGCTGCCTCCCGTAGG |
|  | | SA3 | | AACGAGAGTTTGATCMTGGCTCAG | AACGCTGCTGCCTCCCGTAGG |
|  | | SA5 | | AAGTAGAGTTTGATCMTGGCTCAG | AAGTCTGCTGCCTCCCGTAGG |
|  | | SA7 | | AAGGAGAGTTTGATCMTGGCTCAG | AAGGCTGCTGCCTCCCGTAGG |
|  | | SA9 | | ATATAGAGTTTGATCMTGGCTCAG | ATATCTGCTGCCTCCCGTAGG |
|  | | SA11 | | ATAGAGAGTTTGATCMTGGCTCAG | ATAGCTGCTGCCTCCCGTAGG |
| **Lake Shira** | | SA12 | | ATTTAGAGTTTGATCMTGGCTCAG | ATTTCTGCTGCCTCCCGTAGG |
|  | | SA14 | | ATGTAGAGTTTGATCMTGGCTCAG | ATGTCTGCTGCCTCCCGTAGG |
|  | | SA15 | | ATTGAGAGTTTGATCMTGGCTCAG | ATTGCTGCTGCCTCCCGTAGG |
|  | | SA16 | | ATGCAGAGTTTGATCMTGGCTCAG | ATGCCTGCTGCCTCCCGTAGG |
|  | | SA17 | | ATCTAGAGTTTGATCMTGGCTCAG | ATCTCTGCTGCCTCCCGTAGG |
|  | | SA19 | | ATCCAGAGTTTGATCMTGGCTCAG | ATCCCTGCTGCCTCCCGTAGG |
|  | | SA21 | | ATCGAGAGTTTGATCMTGGCTCAG | ATCGCTGCTGCCTCCCGTAGG |
|  | | SA23 | | ATGAAGAGTTTGATCMTGGCTCAG | ATGACTGCTGCCTCCCGTAGG |
|  | | SN1 | | AAACAGAGTTTGATCMTGGCTCAG | AAACCTGCTGCCTCCCGTAGG |
|  | | SN2 | | AAAGAGAGTTTGATCMTGGCTCAG | AAAGCTGCTGCCTCCCGTAGG |
|  | | SN3 | | AATAAGAGTTTGATCMTGGCTCAG | AATACTGCTGCCTCCCGTAGG |
| **Lake Shunet** | | SN4 | | AATTAGAGTTTGATCMTGGCTCAG | AATTCTGCTGCCTCCCGTAGG |
|  | | SN5 | | AATCAGAGTTTGATCMTGGCTCAG | AATCCTGCTGCCTCCCGTAGG |
|  | | SN5.5 | | AATGAGAGTTTGATCMTGGCTCAG | AATGCTGCTGCCTCCCGTAGG |
|  | | SN6 | | AACAAGAGTTTGATCMTGGCTCAG | AACACTGCTGCCTCCCGTAGG |
|  | | OG0 | | ACATAGAGTTTGATCMTGGCTCAG | ACATCTGCTGCCTCCCGTAGG |
|  | | OG1 | | ACACAGAGTTTGATCMTGGCTCAG | ACACCTGCTGCCTCCCGTAGG |
|  | | OG2 | | ACAGAGAGTTTGATCMTGGCTCAG | ACAGCTGCTGCCTCCCGTAGG |
|  | | OG3 | | ACTAAGAGTTTGATCMTGGCTCAG | ACTACTGCTGCCTCCCGTAGG |
|  | | OG4 | | ACTTAGAGTTTGATCMTGGCTCAG | ACTTCTGCTGCCTCCCGTAGG |
|  | | OG5 | | ACTCAGAGTTTGATCMTGGCTCAG | ACTCCTGCTGCCTCCCGTAGG |
| **Lake Oigon** | | OG6 | | ACTGAGAGTTTGATCMTGGCTCAG | ACTGCTGCTGCCTCCCGTAGG |
|  | | OG7 | | ACCAAGAGTTTGATCMTGGCTCAG | ACCACTGCTGCCTCCCGTAGG |
|  | | OG7.75 | | ACCTAGAGTTTGATCMTGGCTCAG | ACCTCTGCTGCCTCCCGTAGG |
|  | | OG8 | | ACCCAGAGTTTGATCMTGGCTCAG | ACCCCTGCTGCCTCCCGTAGG |
|  | | OG8.25 | | ACCGAGAGTTTGATCMTGGCTCAG | ACCGCTGCTGCCTCCCGTAGG |
|  | | OG8.5 | | ACGAAGAGTTTGATCMTGGCTCAG | ACGACTGCTGCCTCCCGTAGG |
|  | | OG8.75 | | ACGTAGAGTTTGATCMTGGCTCAG | ACGTCTGCTGCCTCCCGTAGG |
|  | | OG8.85 | | ACGCAGAGTTTGATCMTGGCTCAG | ACGCCTGCTGCCTCCCGTAGG |
|  | | OG9 | | ACGGAGAGTTTGATCMTGGCTCAG | ACGGCTGCTGCCTCCCGTAGG |
