# Supplementary material for: Bacterial Communities of Three Saline Meromictic Lakes in Central Asia
Source: PLoS One. 2016 Mar 2;11(3):e0150847. doi: 10.1371/journal.pone.0150847 (PMC4775032; doi:10.1371/journal.pone.0150847)
Supplement: S3 Table — (DOCX) [file pone.0150847.s008.docx]

**S3 Table.** Estimated diversity indices and richness for the bacterial communities as represented in 16S rRNA gene libraries*^a^*.

| *N^b^ S^c^ E^d^ R^e^*  *H^f^* Simpson Chao1 Ace Coverage | | | | | | | | | | |
| --- | --- | --- | --- | --- | --- | --- | --- | --- | --- | --- |
| **Lake Shira** | | | | | | | | | | |
|  | SR1 | 2427 | 182 | 0.74 | 20.68 | 3.87 | 0.04 | 286 | 334 | 0.97 |
|  | SR3 | 1637 | 168 | 0.7 | 23.02 | 3.58 | 0.07 | 267 | 265 | 0.95 |
|  | SR5 | 1528 | 151 | 0.71 | 21.36 | 3.55 | 0.06 | 238 | 350 | 0.96 |
| **Oxic** | SR7 | 1876 | 144 | 0.66 | 19.55 | 3.28 | 0.09 | 274 | 312 | 0.97 |
|  | SR9 | 4442 | 366 | 0.69 | 43.32 | 4.08 | 0.04 | 572 | 718 | 0.96 |
|  | SR11 | 5844 | 408 | 0.65 | 43.8 | 3.90 | 0.07 | 644 | 749 | 0.97 |
|  | SR12 | 4557 | 408 | 0.72 | 44.82 | 4.32 | 0.04 | 601 | 615 | 0.96 |
|  | SR14 | 3638 | 296 | 0.69 | 36.79 | 3.94 | 0.05 | 518 | 601 | 0.96 |
|  | SR15 | 4036 | 280 | 0.69 | 30.23 | 3.87 | 0.06 | 416 | 513 | 0.97 |
|  | SR16 | 3323 | 313 | 0.71 | 40.89 | 4.09 | 0.04 | 496 | 727 | 0.96 |
| **Anoxic** | SR17 | 2997 | 229 | 0.69 | 30.2 | 3.73 | 0.06 | 421 | 518 | 0.97 |
|  | SR19 | 2276 | 178 | 0.68 | 24.13 | 3.54 | 0.06 | 301 | 380 | 0.96 |
|  | SR21 | 4148 | 339 | 0.73 | 39.25 | 4.23 | 0.04 | 560 | 635 | 0.97 |
|  | SR23 | 2682 | 206 | 0.61 | 26.25 | 3.25 | 0.13 | 320 | 411 | 0.97 |
| **Lake Shunet** | | | | | | | | | | |
|  | SN1 | 6979 | 903 | 0.74 | 123.06 | 5.05 | 0.03 | 1650 | 2353 | 0.93 |
| **Oxic** | SN2 | 9465 | 1279 | 0.77 | 172.28 | 5.53 | 0.01 | 2398 | 3587 | 0.93 |
|  | SN3 | 7822 | 1070 | 0.76 | 148.97 | 5.29 | 0.02 | 2079 | 3098 | 0.93 |
|  | SN4 | 6894 | 839 | 0.76 | 110.72 | 5.12 | 0.02 | 1535 | 2143 | 0.94 |
|  | SN5.0 | 7145 | 971 | 0.76 | 129.74 | 5.24 | 0.02 | 1769 | 2459 | 0.93 |
| **Anoxic** | SN5.5 | 8326 | 983 | 0.7 | 127.03 | 4.80 | 0.04 | 1714 | 2328 | 0.94 |
|  | SN6 | 7715 | 890 | 0.7 | 119.36 | 4.76 | 0.04 | 1701 | 2317 | 0.94 |
| **Lake Oigon** | | | | | | | | | | |
|  | OG0 | 1523 | 215 | 0.75 | 35.82 | 4.01 | 0.04 | 397 | 608 | 0.92 |
|  | OG1 | 1194 | 226 | 0.73 | 36.07 | 3.95 | 0.08 | 356 | 506 | 0.91 |
|  | OG2 | 1509 | 204 | 0.68 | 31.14 | 3.64 | 0.07 | 354 | 340 | 0.93 |
| **Oxic** | OG3 | 1330 | 200 | 0.69 | 32.01 | 3.66 | 0.11 | 353 | 501 | 0.92 |
|  | OG4 | 1002 | 125 | 0.61 | 23.99 | 2.95 | 0.15 | 263 | 532 | 0.93 |
|  | OG5 | 1655 | 218 | 0.69 | 32.31 | 3.73 | 0.07 | 354 | 375 | 0.94 |
|  | OG6 | 1311 | 247 | 0.77 | 44.26 | 4.23 | 0.04 | 499 | 744 | 0.89 |
|  | OG7.00 | 2784 | 389 | 0.72 | 57.77 | 4.28 | 0.05 | 732 | 1022 | 0.93 |
|  | OG7.75 | 1398 | 277 | 0.77 | 50.55 | 4.35 | 0.03 | 573 | 814 | 0.89 |
|  | OG8.00 | 831 | 191 | 0.8 | 34.59 | 4.21 | 0.03 | 334 | 484 | 0.88 |
| **Anoxic** | OG8.25 | 1570 | 239 | 0.74 | 43.18 | 4.07 | 0.04 | 623 | 855 | 0.91 |
|  | OG8.50 | 838 | 247 | 0.83 | 47.55 | 4.56 | 0.03 | 463 | 678 | 0.83 |
|  | OG8.75 | 1415 | 250 | 0.76 | 40.31 | 4.19 | 0.04 | 395 | 625 | 0.91 |
|  | OG8.85 | 1067 | 328 | 0.86 | 61.09 | 5.00 | 0.02 | 679 | 919 | 0.83 |
|  | OG9 | 1419 | 331 | 0.81 | 55.2 | 4.71 | 0.02 | 548 | 882 | 0.88 |

*^a^*Calculations were based on OTUs formed at evolutionary distance of <0.03(or~97% similarity).

*^b^N*= the number of sequences.

*^c^S* = the number of OTUs.

*^d^*Evenness= Shannon/In(the number of OTUs)

*^e^*Richness= (number of singleton OTUs-1)/log_10_N. The maximum value is (N-1)/log_10_N.

*^f^H=*Shannon diversity index
